# Therapeutic Applications of Biostable Silver Nanoparticles Synthesized Using Peel Extract of *Benincasa hispida*: Antibacterial and Anticancer Activities

**DOI:** 10.3390/nano10101954

**Published:** 2020-09-30

**Authors:** Wafaa E. Soliman, Salman Khan, Syed Mohd Danish Rizvi, Afrasim Moin, Heba S. Elsewedy, Amr S. Abulila, Tamer M. Shehata

**Affiliations:** 1 Department of Biomedical Sciences, College of Clinical Pharmacy, King Faisal University, Alhofuf, Al-Ahsa 36362, Saudi Arabia; 2 Department of Microbiology and Immunology, Faculty of Pharmacy, Delta University for Science and Technology, Gamasa, Mansoura 11152, Egypt; 3 Department of Biosciences, Integral University, Lucknow 226026, U.P., India; salmank@iul.ac.in; 4 Department of Pharmaceutics, College of Pharmacy, University of Hail, Hail 2240, Saudi Arabia; sm.danish@uoh.edu.sa (S.M.D.R.); a.moinuddin@uoh.edu.sa (A.M.); a.abulila@uoh.edu.sa (A.S.A.); 5 Department of Pharmaceutical Sciences, College of Clinical Pharmacy, King Faisal University, Alhofuf, Al-Ahsa 36362, Saudi Arabia; helsewedy@kfu.edu.sa (H.S.E.); tshehata@kfu.edu.sa (T.M.S.)

**Keywords:** antibacterial, anticancer, *Benincasa hispida*, human cervical cancer cell line, silver nanoparticles

## Abstract

The purpose of this study was to fabricate biostable inorganic silver nanoparticles (AgNPs) using fresh peel (aqueous) extract of *Benincasa hispida*. A fast, robust, and eco-friendly approach was used for the synthesis of AgNPs, where bioactive components of peel extract of *B. hispida* acted as reducing and stabilizing agents. Synthesized AgNPs were characterized using a UV–Vis spectrophotometer, Fourier-transform infrared spectroscopy (FTIR), dynamic light scattering (DLS), and electron microscopy. The synthesized nanoparticles exhibited maximum absorption at 418 nm under the typical AgNPs surface plasmon resonance band range. They depicted a mean size of 26 ± 2 nm with a spherical shape. Their therapeutic prospective was determined by evaluating their antimicrobial and anticancer potential. The bio-synthesized silver nanoparticles exhibited strong antimicrobial activity with minimum inhibitory concentration (MIC 50) values of 14.5, 8.6, 6.063, and 13.4 μg/mL against *Staphylococcus aureus* (ATCC 25923), *Micrococcus luteus* (ATCC 14593), *Escherichia coli* (ATCC 25922), and *Klebsiella pneumonia* (ATCC 13883), respectively. The biosynthesized AgNPs showed potent in vitro cytotoxicity against human cervical cancer cell line with a half maximal inhibitory concentration (IC_50_) value of 0.066 μg/mL; however, no cytotoxic effect was observed on normal human primary osteoblasts cell line. This study explored *B. hispida* extract and confirmed its effectiveness as a promising source in producing AgNPs that could be employed for several therapeutic applications.

## 1. Introduction

In the group of inorganic metals, the use of silver (Ag) can be traced to the Neolithic era. Moyer first recorded the therapeutic use of Ag during the eighth century [1]. In recent times, the tunable photophysical attributes of silver nanoparticles (AgNPs) [2,3,4], their competent addressability by spectroscopic and optical techniques, and speedy advances in nanocrystallizations and fabrication [5] have brought these AgNPs to the forefront of nanoscience research ranging from photonics [6,7] to biomedicine, and clinical [8,9,10,11,12]. Ag has exhibited broad rational use in the medication field since the Archaic period due to its intrinsic nonhazardous characteristics [13,14]. Ag nanomaterial dressings have wound healing properties and have been used as marketable antimicrobial dressings since 1998 [15,16].

AgNPs are known for their strong antimicrobial ability or toxic effects against G + and G − bacteria and protozoa, fungi, and viral infection. The drug-resistance perseverance in micro-organisms has demonstrated the usefulness of Ag, Ag-based compounds, and AgNPs for their antimicrobial features [16]. Ag, Ag + ions, and Ag compounds have antibacterial and antiviral properties [17,18,19]. In small amounts, Ag is nontoxic to normal human cells, but its catalytic oxidation and reaction with dissolved monovalent Ag + ion probably enhance the toxic effect. Elechiguerra et al. [20] demonstrated AgNPs’ size-reliant toxicity on human immunodeficiency virus (type 1) by adhering with glycoproteins (gp120).

Metallic nanoparticles (NPs) mechanistic aspects against pathogenic microbes are important for achieving the synergistic effects with natural compounds. In a broad spectrum, nanoparticles demonstrate the cytotoxicity by releasing reactive oxygen species (ROS) [21,22]; however, their gradual oxidation and the release of Ag+ ions show them to be the strongest bactericidal candidate. Furthermore, their tunable size and easy permeation across the cell membrane trigger interruption of intracellular processes such as cell metabolism, protein synthesis, and cell permeability, which finally lead to cell death. It was verified that the outstanding antimicrobial or bactericidal properties exhibited by the AgNPs are due to their shape, size, morphology, distribution, stability, surface modification or functionalization, and maximum interaction with their environment [8].

Over the past few years, several methods have been introduced for the synthesis of metallic nanoparticles, including chemical reduction. Sodium borohydride or sodium citrate [23] are often used in chemical reduction methods because they are easy to use and economical [24]. The adsorption of toxic chemicals (organic solvents and reducing agents) on the surface of the nanomaterial has produced adverse effects on human health or its applications [25]. Consequently, the use of environmentally-friendly methods of preparation is more desirable. The problem of toxicity can be solved using green machinery like plants, natural compounds, metabolites, and micro-organisms to synthesize metallic nanoparticles [26]. Several biological agents (e.g., alkaloids, phenolic compounds, and terpenoids) and coenzymes were used as stabilizing and reducing agents in the metal nanoparticle formation [27]. The green synthesis of AgNPs was carried out using many herbs followed by an antimicrobial assessment, such as the application of fresh alcoholic green extracts of *Cardiospermum halicacabum* L. leaves [28].

*Benincasa hispida* is commonly known as ash gourd, winter gourd, wax gourd, winter melon, and white pumpkin, and belongs to the cucurbitaceous family. It is a trendy vegetable crop, especially among Asian communities (it originated in the subcontinent of southeast Asia) both for dietary and therapeutic purposes [29,30]. *B. hispida* is widely used as a vegetable and for making candy. It is especially valued as a brain tonic in the treatment of mental disorders and as an effective antidote for alcohol and mercury poisoning [31]. Sarangdhara Samhitaa (an Ayurveda medicine book) describes its use in treating hemorrhage (especially in ulceration of lungs) and pulmonary complications. Its juice from the cortical portion can be used to treat diabetes [32]. Overall, this medicinal plant could be used therapeutically for different health problems such as upper and lower respiratory diseases, gastrointestinal problems, diabetes mellitus, cardiac diseases, and urinary infections. Moreover, its fruits are used as laxative materials, diuretics, and cardio tonics. They have beneficial effects on mental illness, blood disease or infection, jaundice, menstrual disorders, epilepsy, schizophrenia, and other psychological disorders. Phytochemical analysis revealed that the plant’s major constituents include “volatile oils, phenols, flavonoids, saccharides, glycosides, ß-sitosterin, carotenes, proteins, fatty acids, vitamins, uronic acids, minerals, etc.” [33].

In this study, *B. hispida* aqueous peel extract was used to synthesize biostable spherical AgNPs that were further screened for their antibacterial potential against *Staphylococcus aureus, Micrococcus luteus, Escherichia coli, and Klebsiella pneumonia*. The biosynthesized AgNPs’ cytotoxicity was evaluated on human cervical cancer cell line (HeLa) and normal human primary osteoblasts cell line. The AgNPs were characterized by UV–Vis spectroscopy, dynamic light scattering (DLS), FTIR, and electron microscopy. We report the potential of *B. hispida*-synthesized AgNPs for different therapeutic applications given its lesser side effects.

## 2. Materials and Methods

### 2.1. Materials

The solvents, silver nitrate (AgNO_3_), and chemicals were procured from Merck and Sigma-Aldrich. Media for microbiology experiments were obtained from HIMEDIA Laboratories (Mumbai, India). *B. hispida* peel was acquired from the industrial waste of local sweet industries. Dulbecco’s modified Eagle’s medium (DMEM) and fetal bovine serum (FBS) were obtained from Sigma-Aldrich (St. Louis, MO, USA). 3-(4,5-dimethylthiazol-2-yl)-2,5-diphenyl tetrazolium bromide dye (MTT reagent) was purchased from Loba Chemie (Mumbai, India). All other reagents were of the highest grade available.

### 2.2. B. hispida (Aqueous) Peel Extracts Preparation 

The peel of *B. hispida* was washed with tap water followed by distilled water until all the impurities were removed. Then, 20 g of peel was weighed and crushed separately in 50 mL of double distilled water with pestle mortar placed in the polypropylene molded tray filled with ice cubes to prevent denaturation of the proteins. Whatman filter paper (42) was used to filter this mixture before centrifuging it at 6000 rpm for 15 min (4 °C). Pellet was removed by collecting the supernatant in another centrifuge tube, and then the filtered peel extract was stored at cooling temperature for the synthesis of AgNPs. 

### 2.3. Biosynthesis of AgNPs

**Figure 1:**
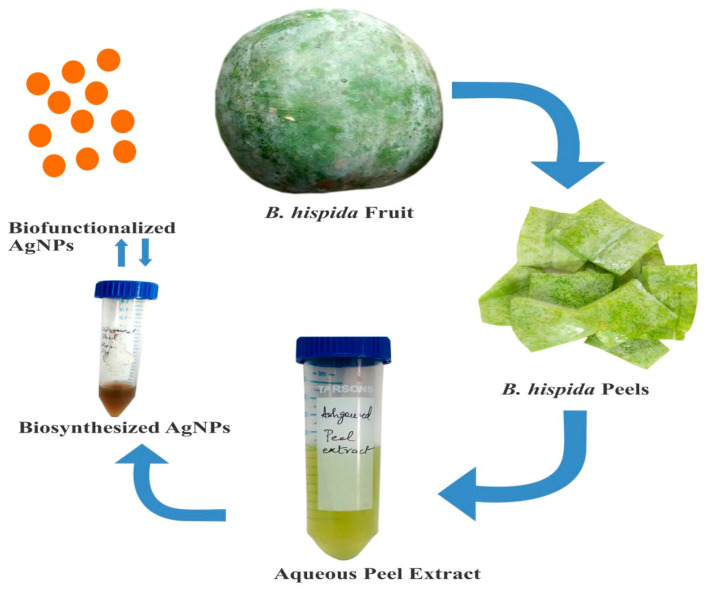
Schematic representation of silver nanoparticles (AgNPs) synthesis.

Initially, 1 M AgNO_3_ stock solution in double-distilled water was prepared before preparing working 1 mM AgNO_3_ solution. Filtered aqueous peel extract of *B. hispida* was mixed with this AgNO_3_ solution in equal ratio until 30 mL volume was reached in a 50 mL screw-capped tube and kept at 40 °C for 5 h. After incubation, the aqueous peel extract changed color from light green to reddish-brown and then was filtered with a 2 µm syringe filter. Absolute ethanol in twice the volume of the filtrate was used to precipitate and remove unbound proteins via centrifugation, followed by filtration. The filtered reaction mixture was kept at a cool temperature until characterization, antibacterial screening, and cytotoxicity assessment on cell lines. A systemic view of AgNPs synthesis is depicted in Figure 1.

### 2.4. Characterization of AgNPs

#### 2.4.1. UV–Visible Spectroscopic Profile of Synthesized AgNPs

One of the most basic and essential techniques for the characterization of nanoparticles is UV–Vis spectroscopy performed by a UV–Vis spectrophotometer (UV-2400PC Series, Shimadzu, Japan). This technique exploits the color-changing property due to the reduction of metal salts to biosynthesized nanoparticles, thereby resulting in surface plasmon resonance (SPR), which can be analyzed and recorded by the UV–Vis spectrometer in the wavelength range of 200–800 nm. The graphs were prepared with Origin-Pro 8.5 software (Version 85E, OriginLab Corporation, Northampton, MA, USA) to accentuate the raw results of the UV–Vis spectroscopy [34].

#### 2.4.2. Transmission Electron Microscopy (TEM)

TEM (TecnaiTM G2 Spirit BioTWIN, FEI, Hillsboro, OR, USA) was applied to depict the shape and size of synthesized AgNPs, and a single drop of AgNPs suspension was dried out on TEM copper grids before performing TEM analysis at 80 kV accelerating voltage [35].

#### 2.4.3. Particle Size and Zeta Potential

Dynamic light scattering (DLS) analysis was used to determine the hydrodynamic radius by approximating the particle size. Both particle size and zeta potential of synthesized AgNPs were determined utilizing Zetasizer Nano-ZS (ZEN3600 Malvern Instrument Ltd., Malvern, UK) [36]. 

#### 2.4.4. FTIR

To observe the functional group present on the synthesized AgNPs surface, FTIR analysis was performed using FTIR spectroscopy (PerkinElmer Inc., Waltham, MA, USA) via a full reflectance sampling tool and scanning by applying a transmission technique with above a 4000–650 cm^−1^ range wave number of 4 cm^−1^ resolutions [37].

### 2.5. Antibacterial Activity of AgNPs

Antibacterial assessment of synthesized AgNPs was performed primarily by the disk diffusion technique [38]. However, after the selection of active AgNPs based on preliminary results, minimum inhibitory concentration (MIC) was evaluated by applying the microbroth dilution approach on the microtiter plate [39]. *Escherichia coli* (ATCC 25922), *Staphylococcus aureus* (ATCC 25923), *Klebsiella pneumoniae* (ATCC 13883), and *Micrococcus luteus* (ATCC 14593) bacterial strains were procured from the National Chemical Laboratory, India. Mid-log phase bacteria for each strain were harvested through centrifugation and washed with Na-PO_4_ buffer before dilution to the desired concentration in a Luria–Bertani (LB) broth. Serial dilutions of AgNPs were performed with LB broth to attain the anticipated concentrations of 0.1 to 20 μg⁄mL in microtiter plates, and 5 × 10^4^ colony-forming unit (CFU) of bacterial inoculum was added to each. MIC was determined as the minimum AgNPs concentration where growth inhibition was observed after overnight incubation of the microtiter plates at 37 °C.

### 2.6. Analysis of Cytomorphological Changes and Cytotoxicity in HeLa and Primary Osteoblasts

#### 2.6.1. Cell Culture

The human cervical cancer cell lines (HeLa) and normal human primary osteoblasts cell lines were procured from the National Centre for Cell Science (NCCS), Pune, India. HeLa and primary osteoblast cells were grown as a monolayer in DMEM and Mac Coy’s media, respectively, supplemented with 10% fetal bovine serum and 1% antibiotic. The cell lines were maintained and grown at 37 °C with a humidified atmosphere containing 5% of CO_2_. 

#### 2.6.2. Assessment of Cytomorphological Changes 

Various concentrations of synthesized AgNPs were added to HeLa and human primary osteoblasts cells and incubated at 37 °C for 48 h (5% CO_2_). An inverted phase-contrast microscope (Nikon ECLIPSE Ti-S, Tokyo, Japan) was used to perceive the gross changes in morphology after incubation.

#### 2.6.3. Assessment of Cytotoxicity

Initially, both the cancerous and normal cell lines (1 × 10^4^ cells/well) were added to a 96-well microtiter plate and incubated at 37 °C for 24 h. After incubation, various concentrations, i.e., 0.363, 0.176, 0.0922, 0.036, 0.0222, and 0.0162 µg/mL, of synthesized AgNPs were added in triplicates and incubated at 37 °C for 48 h. Then, the cytotoxicity was checked by 3-(4, 5-dimethylthiazol-2-yl)-2, 5-diphenyl-tetrazolium bromide (MTT) cytotoxicity assay. The media of 96-well plates were discarded, followed by the addition of 50 µL of MTT dye (prepared at 5 mg/mL in phosphate buffer saline (PBS)) in each well and incubation at 37 °C for 4 h. Dimethyl sulfoxide (DMSO; 150 µL) was added to dissolve the formazan crystals. A microplate reader (BIORAD-680) was used to estimate the amount of reduced MTT by measuring the optical density (OD) at 570 nm with a reference filter of 655 nm [40]. Cell growth inhibition percentage was evaluated using the formula 100 − (A_test_ − A_blank_)/(A_control_ − A_blank_) × 100, where test absorbance is A_test_, blank absorbance is A_blank,_, and control absorbance is A_control_. 

## 3. Results and Discussion

### 3.1. Biosynthesis and Characterization of AgNPs

The AgNPs were synthesized by incubating 15 mL of B. hispida aqueous peel extract with 1 mM of AgNO_3_ solution. We found that aqueous peel extract could synthesize AgNPs due to its reducing enzymes and capping agents like secondary metabolites that could synergistically reduce AgNO_3_ (+1) oxidation state to Ag (0) oxidation state. The reaction mixture performed under identical control conditions in the absence of the aqueous peel extract did not show any changes in absorption, signifying the role of aqueous peel extract in the formation of AgNPs. Similarly, the incubation of the aqueous peel extract alone in double distilled water did not show any absorption peak(s) characteristic of AgNPs. A related approach for AgNPs biosynthesis was applied by using Mentha piperita leaf extract in 2018 [39,41]. 

#### 3.1.1. UV–Visible Spectroscopic Profile of Synthesized AgNPs

**Figure 2:**
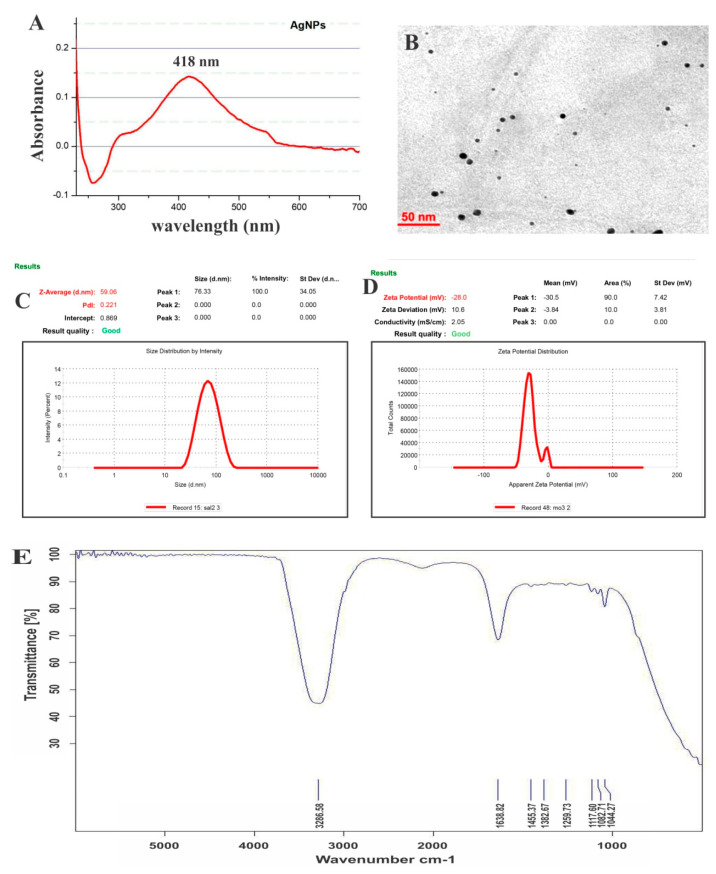
AgNPs characterization by (**A**) UV–visible spectroscopy, (**B**) TEM analysis, (**C**) DLS, (**D**) zeta potential, and (**E**) FTIR spectroscopy.

UV–visible spectroscopic investigation was performed, and a characteristic maximum absorption centered at 418 nm was recorded as presented in Figure 2A, which was attributed to the surface plasmon resonance (SPR) band of the AgNPs. 

#### 3.1.2. TEM

The high-resolution image (Figure 2B) was acquired using the transmission electron microscope (TEM), which confirmed the average size of AgNPs as 26 ± 2 nm using a Gatan digital micrograph, and showed by the spherical form of AgNPs. The TEM micrographs did not expose the agglomeration of the as-synthesized AgNPs. 

#### 3.1.3. Particle Size and Zeta Potential

A dynamic light scattering microscope (DLS) and zeta potential of AgNPs were adopted to characterize the physicochemical characteristics of the prepared nanoparticles. The prepared AgNPs had an average size within the nanosize range (Figure 2C). The zeta potential of the prepared AgNPs was −28 mV (Figure 2D), suggesting higher stability of the particles. The synthesized AgNPs were found to be stable with no aggregation when stored at room temperature. The electrostatic repulsive forces among nanoparticles avoid their agglomeration in aqueous suspension and prevent them from approaching each other. In this study, the DLS-estimated size was larger than the TEM size. DLS estimates the hydrodynamic diameter of the inorganic core and the solvent layer attached to the particle, whereas TEM provides information about the size of the inorganic core alone without the hydration layer. Thus, the size obtained by DLS will always be larger than the actual size estimated by TEM [42,43]. 

#### 3.1.4. FTIR

The FTIR analysis of synthesized AgNPs in Figure 2E depicts an existing peak focused at 1643.24 cm^−1^, i.e., uniqueness of amide C=O groups. A medium and wide shoulder for the amide I linkage and amide II band was observed at 1537.02 cm^−1^. N–H twist and carboxyl stretch in the protein amide bond were found to be responsible for the presence of amides bands I and II that are capped or surface-modified on AgNPs [41]. The N–H stretch vibration peak was observed at 3296.5 cm^−1^; however, this vibration is susceptible to hydrogen bond strength with no dependence on backbone confirmation. Moreover, the alcohol and ether group (C–O–C/C–OH) C–O stretch [41], along with (aliphatic amine) the C–N stretch vibration, showed a peak at 1081.8 cm^−1^. The alkynes C≡C stretched vibration because numerous secondary metabolites showed a peak at 2127.39 cm^−1^. Peaks at 3756.013 and 3868.76 cm^−1^ were observed for free (O–H) hydroxyl on the terminus.

### 3.2. Antibacterial Activity of AgNPs

**Figure 3:**
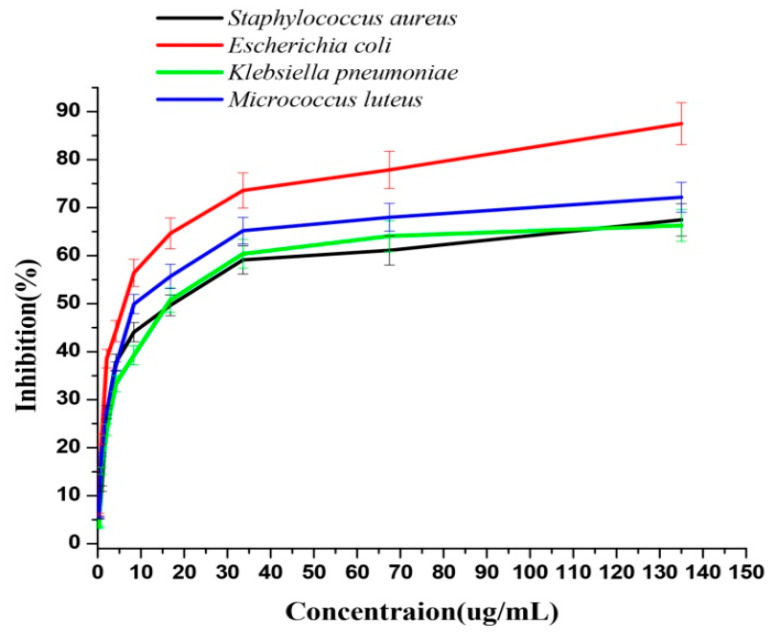
The antibacterial potential of AgNPs against Staphylococcus aureus, Escherichia coli, Klebsiella pneumonia, and M. luteus.

We found that biogenic AgNPs synthesized by B. hispida aqueous peel extract shows a strong antibacterial activity against both Gram-positive and Gram-negative pathogenic bacterial strains. The MIC_50_ of AgNPs (Figure 3) was evaluated against different pathogenic bacterial strains that included 14.5 μg/mL against S. aureus, 8.6 μg/mL against M. luteus, 6.063 μg/mL against E. coli, and 13.4 μg/mL against K. pneumoniae, indicating its broad-spectrum feature. However, we found that AgNPs were more effective against E. coli (Gram negative) and M. luteus (Gram positive) than other pathogenic strains [8]. Similar inhibition behavior was observed when AgNPs were synthesized by leaf extract of Carya illinoinensis [44]. It was proposed by several reports that the lower AgNPs potential against Gram-positive bacteria was due to differences in cell walls [44,45,46,47]. A thick peptidoglycan layer in Gram-positive bacteria prohibited the entry of AgNPs into the cytoplasm, and a higher AgNPs concentration is required to inhibit the growth of Gram-positive than Gram-negative bacteria [44,48].

Silver (Ag) has been applied for countering spoilage and infections since archaic times. The mechanism of inorganic metallic nanoparticles against pathogenic microbes is significant for achieving synergistic effects with natural compounds. In broad-spectrum applications, the hypothetical mechanism of cytotoxicity exhibited by metallic nanoparticles occurs via releasing reactive oxygen species (ROS) [49,50]. However, its slow oxidation and Ag+ ions release reveal it as the strongest biocidal agent or molecule against pathogenic micro-organisms. It was widely reported that the loss of sub-cellular materials because of pit creations in the cell membrane is a feature of the bactericidal action of AgNPs. This action of AgNPs is augmented by the inhibition of respiratory chain dehydrogenases, subsequently affecting cell growth. Some phospholipids and proteins might work jointly to trigger membrane breakdown, causing death and cell decomposition [49]. The pathogenic G+ bacteria react differently after exposure to inorganic metal nanoparticles [23]. The antibacterial mechanism shows that AgNPs attack both primary and secondary (α-helix) cell wall structures by creating a link with the cell wall peptide and glycan, prompting the formation of pits. AgNPs form a link with N-acetylglucosamine and N-acetylmuramic acid (β–1/4 bonds), leading to obliteration of their interaction and liberating them to the surroundings [51]. 

### 3.3. Cytotoxic Effect of AgNPs on HeLa and Primary Osteoblasts

**Figure 4:**
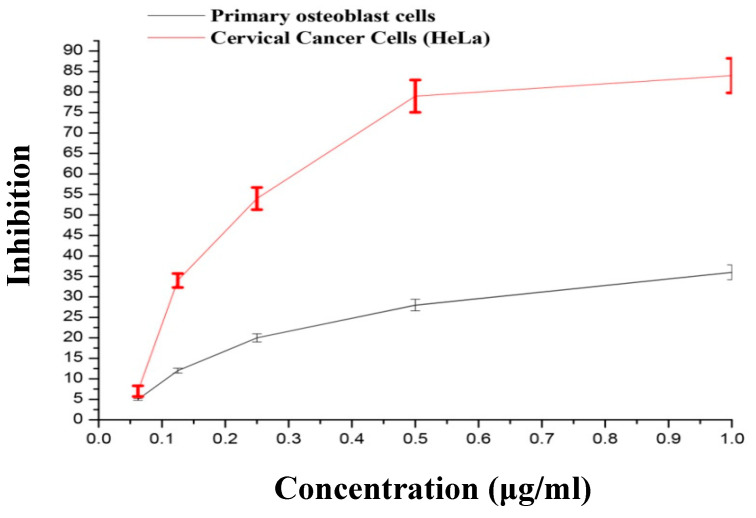
The dose-dependent AgNPs cytotoxicity on HeLa and primary osteoblast cells. The data are shown as mean ± SD of triplicate experiments.

The toxicity of AgNPs was investigated on HeLa and primary osteoblasts cell line using the MTT cell proliferation assessment. The cell viability was screened at different AgNPs concentrations (0.363, 0.176, 0.0922, 0.036, 0.0222, and 0.0162 µg/mL; Figure 4). The outcome showed that HeLa cell lines (Figure 3) quickly lost their viability when incubated with AgNPs with the concentrations range from 0.0116 to 0.156 µg/mL. However, it was not significantly decreased when AgNPs were applied at the increased concentration range of 0.156 to 0.313 µg/mL. These AgNPs showed less toxicity against normal primary osteoblasts cell lines, demonstrating their higher acceptable biological limits. The IC_50_ value obtained in HeLa cells was 0.066 µg/mL. Similarly, Melia-azedarach- and Sargassum-wightii-biosynthesized AgNPs showed dose-dependent cytotoxicity against HeLa cells [52]. However, a marked decrease in glutathione and increased lipid peroxidation, leading to elevated oxidative stress, were suggested as two reasons for AgNPs’ antiproliferative action on HeLa cells [53]. In other studies, selective cytotoxicity against cancer cells was observed for AgNPs synthesized by walnut extract and lignin extracted from wheat [54,55]. The reason for cancer selectivity was attributed to the higher AgNPs uptake by cancerous cells compared to normal cells, presumably due to atypical metabolism and increased rate of proliferation [54,56]. In our study, capping of natural components of *B. hispida* on synthesized AgNPs could be attributed to the synergistic effect on cancer cells and reduced toxicity on a normal cell. It was observed that herbal natural compounds show selective toxicity against abnormal cells [54,57].

**Figure 5:**
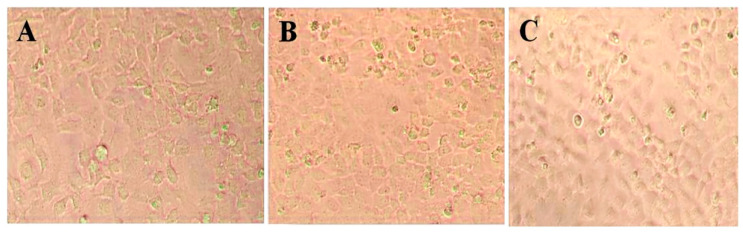
AgNPs cytotoxicity against HeLa cell lines (20 times magnification). Where (**A**) is untreated control HeLa cells, and (**B**,**C**) are HeLa cells treated with AgNPs.

HeLa and primary osteoblast cells with 70% confluence were kept with AgNPs in incubation for 48 h. The phase-contrast microscopy pictures (Figure 5B) revealed alterations in the HeLa cell morphology. The maximum number of HeLa cells (Figure 5C) showed notable shape variations (changing into circular), condensation of cytoplasm, membrane integrity failure, clumping of cells, and inhibition of cell growth. In contrast, insignificant morphological variations were observed in primary osteoblast cells compared to the control untreated normal cells. The synergistic effect of camptothecin and AgNPs on HeLa cells showed an augmented oxidative stress level and expression of the pro-apoptotic gene and increased disruption of membrane permeability as compared with camptothecin alone [58].

The findings of our study suggested that *B. hispida*-biosynthesized AgNPs have broad-spectrum antibacterial potential along with anticancer potential against human cervical cancer cells. However, the outcomes of the study should be explored to develop into multipotent therapeutic agents against different diseases.

## 4. Conclusions

This paper described a green and environmentally-friendly method to produce AgNPs in large amounts. The formation of AgNPs with *B. hispida* aqueous peel extract was observed by the color change of *B. hispida* aqueous peel extract to brownish-yellow. The color changes indicated that AgNPs had produced the desired average nanometer size. *B. hispida* peel extract aqueous solution acted as a reducing and stabilizing agent. These biogenic AgNPs exhibited significant dose-dependent antibacterial and anticancer potentials. However, further investigations are warranted to assess the toxicity details and the mechanism associated with the antibacterial and anticancer action of the biosynthesized AgNPs. Nevertheless, the outcomes of the present study provide a broad AgNPs-based platform for various therapeutic applications in the near future. 

## References

[1] Moyer C. (1965). A treatment of burns. Trans. Stud. Coll. Physicians Phila.

[2] Nie S., Emory S.R. (1997). Probing Single Molecules and Single Nanoparticles by Surface-Enhanced Raman Scattering. Science.

[3] Henglein A. (1993). Physicochemical properties of small metal particles in solution: “Microelectrode” reactions, chemisorption, composite metal particles, and the atom-to-metal transition. J. Phys. Chem..

[4] Huang Y.-F., Chang H.-T., Tan W. (2008). Cancer Cell Targeting Using Multiple Aptamers Conjugated on Nanorods. Anal. Chem..

[5] Shrivas K., Wu H.F. (2008). Modified silver nanoparticle as a hydrophobic affinity probe for analysis of peptides and proteins in biological samples by using liquid-liquid microextraction coupled to AP-MALDI-ion trap and MALDI-TOF mass spectrometry. Anal. Chem..

[6] Schultz S., Smith D.R., Mock J.J., Schultz D.A. (2000). Single-target molecule detection with nonbleaching multicolor optical immunolabels. Proc. Natl. Acad. Sci. USA.

[7] Nair L.S., Laurencin C.T. (2007). Silver nanoparticles: Synthesis and therapeutic applications. J. Biomed. Nanotechnol..

[8] Krutyakov Y.A., Kudrinskiy A.A., Olenin A.Y., Lisichkin G.V. (2008). Synthesis and properties of silver nanoparticles: Advances and prospects. Russ. Chem. Rev..

[9] Tripathi A., Chandrasekaran N., Raichur A., Mukherjee A. (2009). Antibacterial applications of silver nanoparticles synthesized by aqueous extract of Azadirachta indica (Neem) leaves. J. Biomed. Nanotechnol..

[10] Durán N., Marcato P.D., De Souza G.I., Alves O.L., Esposito E. (2007). Antibacterial effect of silver nanoparticles produced by fungal process on textile fabrics and their effluent treatment. J. Biomed. Nanotechnol..

[11] Vigneshwaran N., Kathe A.A., Varadarajan P., Nachane R.P., Balasubramanya R. (2006). Biomimetics of silver nanoparticles by white rot fungus, *Phaenerochaete chrysosporium*. Colloids Surf. B Biointerfaces.

[12] Kumar A., Vemula P.K., Ajayan P.M., John G. (2008). Silver-nanoparticle-embedded antimicrobial paints based on vegetable oil. Nat. Mater..

[13] Pal S., Tak Y.K., Song J.M. (2007). Does the Antibacterial Activity of Silver Nanoparticles Depend on the Shape of the Nanoparticle? A Study of the Gram-Negative Bacterium *Escherichia coli*. Appl. Environ. Microbiol..

[14] Klueh U., Wagner V., Kelly S., Johnson A., Bryers J.D. (2000). Efficacy of silver-coated fabric to prevent bacterial colonization and subsequent device-based biofilm formation. J. Biomed. Mater. Res..

[15] Wright J.B., Lam K., Buret A.G., Olson M.E., Burrell R.E. (2002). Early healing events in a porcine model of contaminated wounds: Effects of nanocrystalline silver on matrix metalloproteinases, cell apoptosis, and healing. Wound Repair Regen..

[16] Bhol K.C., Alroy J., Schechter P.J. (2004). Anti-inflammatory effect of topical nanocrystalline silver cream on allergic contact dermatitis in a guinea pig model. Clin. Exp. Dermatol..

[17] Oka H., Tomioka T., Tomita K., Nishino A., Ueda S. (1994). Inactivation of enveloped viruses by a silver-thiosulfate complex. Met. Based Drugs.

[18] Oloffs A., Grosse-Siestrup C., Bisson S., Rinck M., Rudolph R., Gross U. (1994). Biocompatibility of silver-coated polyurethane catheters and silvercoated Dacron^®^ material. Biomaterials.

[19] Chang T.-W. (1975). Antiherpesviral Activity of Silver Sulfadiazine. J. Cutan. Pathol..

[20] Elechiguerra J.L., Burt J.L., Morones J.R., Camacho-Bragado A., Gao X., Lara H.H., Yacaman M.J. (2005). Interaction of silver nanoparticles with HIV-1. J. Nanobiotechnol..

[21] Sun Y., Mayers B., Xia Y. (2003). Transformation of Silver Nanospheres into Nanobelts and Triangular Nanoplates through a Thermal Process. Nano Lett..

[22] Jee S.C., Kim M., Shinde S.K., Ghodake G.S., Sung J.S., Kadam A.A. (2020). Assembling ZnO and Fe_3_O_4_ nanostructures on halloysite nanotubes for anti-bacterial assessments. Appl. Surf. Sci..

[23] Sondi I., Salopek-Sondi B. (2004). Silver nanoparticles as antimicrobial agent: A case study on *E. coli* as a model for Gram-negative bacteria. J. Colloid Interface Sci..

[24] Zielińska A., Skwarek E., Zaleska A., Gazda M., Hupka J. (2009). Preparation of silver nanoparticles with controlled particle size. Procedia Chem..

[25] Singh A., Jain D., Upadhyay M., Khandelwal N., Verma H. (2010). Green synthesis of silver nanoparticles using Argemone mexicana leaf extract and evaluation of their antimicrobial activities. Dig. J. Nanomater. Bios..

[26] Mulfinger L., Solomon S.D., Bahadory M., Jeyarajasingam A.V., Rutkowsky S.A., Boritz C. (2007). Synthesis and Study of Silver Nanoparticles. J. Chem. Educ..

[27] Chandran S.P., Chaudhary M., Pasricha R., Ahmad A., Sastry M. (2006). Synthesis of gold nanotriangles and silver nanoparticles using Aloe vera plant extract. Biotechnol. Prog..

[28] Shekhawat M.S., Manokari M., Kannan N., Revathi J., Latha R. (2013). Synthesis of silver nanoparticles using Cardiospermum halicacabum L. leaf extract and their characterization. J. Phytopharmacol..

[29] Nimbal S.K., Venkatrao N., Ladde S., Pujar B. (2011). Anxiolytic evaluation of Benincasa hispida (Thunb) Cogn. fruit extracts. Int. J. Pharm. Pharm. Sci. Res..

[30] Zaini N.A.M., Anwar F., Hamid A.A., Saari N. (2011). Kundur [Benincasa hispida (Thunb.) Cogn.]: A potential source for valuable nutrients and functional foods. Food Res. Int..

[31] Arora P., Kaushik D. (2016). Therapeutic potential of Benincasa cerifera: A review. Chin. J. Integr. Med..

[32] Lee K.H., Choi H.R., Kim C.H. (2005). Anti-angiogenic effect of the seed extract of Benincasa hispida Cogniaux. J. Ethnopharmacol..

[33] Al-Snafi A.E. (2013). The Pharmacological importance of Benincasa hispida. A review. Int. J. Pharma Sci. Res..

[34] Hamouda R.A., Hussein M.H., Abo-elmagd R.A., Bawazir S.S. (2019). Synthesis and biological characterization of silver nanoparticles derived from the cyanobacterium *Oscillatoria limnetica*. Sci. Rep..

[35] Rauwel P., Küünal S., Ferdov S., Rauwel E. (2015). A Review on the Green Synthesis of Silver Nanoparticles and Their Morphologies Studied via TEM. Adv. Mater. Sci. Eng..

[36] Elsewedy H.S., Dhubiab B.E.A., Mahdy M.A., Elnahas H.M. (2020). Development, optimization, and evaluation of PEGylated brucine-loaded PLGA nanoparticles. Drug Deliv..

[37] Shehata T.M., Mohafez O.M., Hanieh H.N. (2018). Pharmaceutical Formulation and Biochemical Evaluation of Atorvastatin Transdermal Patches. Indian J. Pharm. Educ. Res..

[38] Ma Y., Liu C., Qu D., Chen Y., Huang M., Liu Y. (2017). Antibacterial evaluation of sliver nanoparticles synthesized by polysaccharides from *Astragalus membranaceus* roots. Biomed. Pharmacother..

[39] Khatoon A., Khan F., Ahmad N., Shaikh S., Rizvi S.M.D., Shakil S., Al-Qahtani M.H., Abuzenadah A.M., Tabrez S., Ahmed A.B.F. (2018). Silver nanoparticles from leaf extract of Mentha piperita: Eco-friendly synthesis and effect on acetylcholinesterase activity. Life Sci..

[40] Deng X., Yin F., Lu X., Cai B., Yin W. (2006). The apoptotic effect of brucine from the seed of Strychnos nux-vomica on human hepatoma cells is mediated via Bcl-2 and Ca_2_^+^ involved mitochondrial pathway. Toxicol. Sci..

[41] Philip D. (2011). Mangifera indica leaf-assisted biosynthesis of well-dispersed silver nanoparticles. Spectrochim. Acta Part A Mol. Biomol. Spectrosc..

[42] Park K., Kittelson D.B., McMurry P.H. (2004). Structural properties of diesel exhaust particles measured by transmission electron microscopy (TEM): Relationships to particle mass and mobility. Aerosol Sci. Technol..

[43] Berne B.J., Pecora R. (2000). Dynamic Light Scattering with Applications to Biology, Chemistry and Physics.

[44] Dalir S.J.B., Djahaniani H., Nabati F., Hekmati M. (2020). Characterization and the evaluation of antimicrobial activities of silver nanoparticles biosynthesized from *Carya illinoinensis* leaf extract. Heliyon.

[45] Loo Y.Y., Rukayadi Y., Nor-Khaizura M.A., Kuan C.H., Chieng B.W., Nishibuchi M., Radu S. (2018). In Vitro Antimicrobial Activity of Green Synthesized Silver Nanoparticles Against Selected Gram-negative Foodborne Pathogens. Front. Microbiol..

[46] Chatterjee T., Chatterjee B.K., Majumdar D., Chakrabarti P. (2015). Antibacterial effect of silver nanoparticles and the modeling of bacterial growth kinetics using a modified Gompertz model. Biochim. Biophys. Acta.

[47] Qing Y., Cheng L., Li R., Liu G., Zhang Y., Tang X., Wang J., Liu H., Qin Y. (2018). Potential antibacterial mechanism of silver nanoparticles and the optimization of orthopedic implants by advanced modification technologies. Int. J. Nanomed..

[48] Malanovic N., Lohner K. (2016). Gram-positive bacterial cell envelopes: The impact on the activity of antimicrobial peptides. Biochim. Biophys. Acta (BBA)-Biomembr..

[49] Li W.-R., Xie X.-B., Shi Q.-S., Zeng H.-Y., You-Sheng O.-Y., Chen Y.-B. (2010). Antibacterial activity and mechanism of silver nanoparticles on *Escherichia coli*. Appl. Microbiol. Biotechnol..

[50] Nel A.E., Mädler L., Velegol D., Xia T., Hoek E.M., Somasundaran P., Klaessig F., Castranova V., Thompson M. (2009). Understanding biophysicochemical interactions at the nano–bio interface. Nat. Mater..

[51] Li W.-R., Xie X.-B., Shi Q.-S., Duan S.-S., Ouyang Y.-S., Chen Y.-B. (2011). Antibacterial effect of silver nanoparticles on Staphylococcus aureus. Biometals.

[52] Sukirtha R., Priyanka K.M., Antony J.J., Kamalakkannan S., Thangam R., Gunasekaran P., Krishnan M., Achiraman S. (2012). Cytotoxic effect of Green synthesized silver nanoparticles using Melia azedarach against in vitro HeLa cell lines and lymphoma mice model. Process Biochem..

[53] Kakade D., Arora S., Ambwani S. (2018). Anti-proliferative effect of silver nanoparticles in HeLa cells due to enhanced oxidative stress. Res. J. Biotechnol..

[54] Khorrami S., Zarrabi A., Khaleghi M., Danaei M., Mozafari M.R. (2018). Selective cytotoxicity of green synthesized silver nanoparticles against the MCF-7 tumor cell line and their enhanced antioxidant and antimicrobial properties. Int. J. Nanomed..

[55] Saratale R.G., Saratale G.D., Ghodake G., Cho S.K., Kadam A., Kumar G., Jeon B.H., Pant D., Bhatnagar A., Shin H.S. (2019). Wheat straw extracted lignin in silver nanoparticles synthesis: Expanding its prophecy towards antineoplastic potency and hydrogen peroxide sensing ability. Int. J. Biol. Macromol..

[56] Cairns R.A., Harris I.S., Mak T.W. (2011). Regulation of cancer cell metabolism. Nat. Rev. Cancer.

[57] Li L., Tsao R., Yang R., Liu C., Zhu H., Young J.C. (2006). Polyphenolic profiles and antioxidant activities of heartnut (Juglans ailanthifolia Var. cordiformis) and Persian walnut (*Juglans regia* L.). J. Agric. Food Chem..

[58] Yuan Y.-G., Zhang S., Hwang J.-Y., Kong I.-K. (2018). Silver nanoparticles potentiates cytotoxicity and apoptotic potential of camptothecin in human cervical cancer cells. Oxidative Med. Cell. Longev..

